# Multicenter Practice of Non/Minimized Fluoroscopy Ablation for Paroxysmal AF in China: The PAF-ICE Trial

**DOI:** 10.1016/j.jacasi.2025.07.011

**Published:** 2025-09-05

**Authors:** Yunhe Wang, Deyong Long, Fangyi Xiao, Minglong Chen, Xingpeng Liu, Jidong Zhang, Yumei Xue, Jie Fan, Haixiong Wang, Mengzuo Wu, Rui Wang, Jia Li, Tao He, Weili Ge, Xiaobo Huang, Ruhong Jiang, Qiang Liu, Zuwen Zhang, Guosheng Fu, Chenyang Jiang

**Affiliations:** a Department of Cardiology, Sir Run Run Shaw Hospital, Zhejiang University School of Medicine, Hangzhou, China; b Department of Cardiology, Beijing Anzhen Hospital, Capital Medical University, Beijing, China; c Department of Cardiology, The First Affiliated Hospital of Wenzhou Medical University, Wenzhou, China; d Department of Cardiology, The First Affiliated Hospital of Nanjing Medical University, Nanjing, China; e Department of Cardiology, Beijing Chaoyang Hospital, Capital Medical University, Beijing, China; f Department of Cardiology, Second Hospital of Hebei Medical University, Shijiazhuang, China; g Department of Cardiology, Guangdong Provincial People's Hospital, Guangzhou, China; h Department of Cardiology, Yunnan First People's Hospital, Kunming, China; i Department of Cardiology, Cardiovascular Hospital of Shanxi Province, Taiyuan, China; j Department of Cardiology, The First Affiliated Hospital of Anhui Medical University, Hefei, China; k Department of Cardiology, First Hospital of Shanxi Medical University, Taiyuan, Shanxi Province, China; l Department of Cardiology, The Second Affiliated Hospital of Wenzhou Medical University, Wenzhou, China; m Department of Cardiology, Sichuan Provincial People's Hospital, Chengdu, China; n Department of Cardiology, Taizhou Hospital of Zhejiang Province, Taizhou, China; o Department of Cardiology, Nanfang Hospital, Southern Medical University, Guangzhou, China; p Comprehensive Unit of National Regional Medical Center, Zhejiang Province, China

**Keywords:** atrial fibrillation, catheter ablation, intracardiac echocardiography, zero fluoroscopy

## Abstract

**Background:**

Intracardiac echocardiography (ICE)-guided non/minimized-fluoroscopy catheter ablation for atrial fibrillation (AF) has been reported, but its effectiveness and safety still lack multicenter evidence.

**Objectives:**

The authors sought to evaluate the effectiveness and safety of ICE-guided non/minimized-fluoroscopy catheter ablation compared with the traditional fluoroscopy-guided approach in patients with paroxysmal AF.

**Methods:**

A total of 448 patients with paroxysmal AF, from 15 centers in China, were randomly assigned in a 1:1 ratio to a non/minimized-fluoroscopy group (n = 223) and a traditional approach group (n = 225). The primary efficacy endpoint was freedom from AF recurrence after a single ablation procedure. The primary safety endpoint was a composite of death from any cause, stroke or transient ischemic attack, and other serious adverse events.

**Results:**

Pulmonary vein isolation was achieved in all patients. After a median follow-up of 12.2 (Q1-Q3: 8.8-17.7) months, 184 of 223 patients (82.5%) in the non/minimized-fluoroscopy group and 189 of 225 (84.0%) in the traditional approach group remained free from arrhythmia. Cox analysis showed a HR of 0.949 (95% CI: 0.774 to 1.164); *P* = 0.858, demonstrating the noninferiority of the non/minimized-fluoroscopy approach. The primary safety endpoint did not differ significantly in the 2 groups (*P* = 0.975). This protocol enabled near zero–radiation procedures (mean <1 mGy) in 7 of 15 centers (46.7%), and radiation-free AF ablation in 125 of 223 patients (56.1%), significantly reducing x-ray exposure and operator radiation protection equipment usage.

**Conclusions:**

ICE-combined non/minimized-fluoroscopy AF ablation was noninferior in effectiveness compared to traditional AF ablation, with no significant difference in safety endpoints, indicating its potential of widespread adoption.

Catheter ablation for atrial fibrillation (AF) has proven to be an effective treatment for symptom relief and a reduction in AF burden.^1^ The utilization of AF ablation has markedly increased over the last decade. Owing to technology such as 3-dimensional (3D) electroanatomical mapping (EAM), reliance on x-rays for cardiac catheter ablation procedures has gradually decreased. However, many electrophysiology centers run busy, daily, conventional procedure programs and, although single doses of radiation are not large, accumulated radiation remains of concern. Indeed, cumulative radiation exposure can cause numerous health problems for both patients and electrophysiology (EP) lab staff.^2^^,^^3^ Current interventional procedures all follow the ALARA (as low as reasonably achievable) principle,^4^ to minimize radiation exposure. In addition, medical personnel wearing protective gear such as lead aprons may experience back pain and suffer spinal problems, potentially shortening their career lifespan.^5^, ^6^, ^7^ Currently, AF catheter ablation procedures constitute the main daily workload of most EP labs. As awareness and diagnosis of AF gradually increases, the volume of these procedures is expected to grow further in the future. A series of clinical studies have shown that radiation-free or extremely low-radiation AF ablation under intracardiac echocardiography (ICE) guidance is technically feasible, and it has already been adopted by experienced centers.^8^, ^9^, ^10^, ^11^, ^12^, ^13^, ^14^ However, evidence of its efficacy and safety in multicenter practice is required.

## Methods

The PAF-ICE trial (ICE-Guided Minimal-Fluoroscopy Ablation in Patients with PAF Trial; ChiCTR2000033624) was a noninferior, multicenter, prospective randomized controlled trial comparing the efficacy and safety of ICE-combined non/minimized-fluoroscopy ablation with a traditional approach in patients with paroxysmal AF. The trial design has been published previously.^15^ The study also aimed to determine whether this non/minimized protocol can be successfully applied nationwide.

### Patient population

A total of 448 patients were recruited from 15 centers in China. Patients were eligible if they were 18 to 75 years old, had symptomatic paroxysmal AF, and were undergoing ablation for the first time. Written informed consent was obtained from all patients before trial procedures. The ethics review for this study was approved by the Ethics Committee of Sir Run Run Shaw Hospital, Zhejiang University School of Medicine, which served as the initiating center. Ethics approvals were also obtained from the ethics committees of the other 14 participating centers in accordance with their respective institutional requirements. Exclusion criteria included patients with myocardial infarction or coronary revascularization in the previous 3 months, NYHA functional class grading III or IV, left atrial diameter >50 mm, left ventricular ejection fraction <40%, or thrombus in the left atrium/appendage.

### Study procedure

All antiarrhythmic drugs except amiodarone were discontinued for at least 5 half-lives before ablation. Transesophageal echocardiography, left atrial enhancement computed tomography, or ICE was performed to screen the left atrium/appendage for thrombus, and oral anticoagulation therapy was not interrupted during the periprocedure period. All procedures were performed under conscious sedation. The activated clotting time was maintained between 250 and 350 seconds.

Patients were randomly assigned in a 1:1 ratio to the non/minimized-fluoroscopy or the traditional approach group.

### Non/minimized-fluoroscopy group

An ICE catheter (SOUNDSTAR, Biosense Webster) was advanced into the right atrium. Patients demonstrating thrombus in the left atrium/appendage were excluded before transseptal puncture. Pericardial effusion was quantitatively assessed at baseline. Geometries including the right atrium, coronary sinus (CS), left atrium, pulmonary veins (PVs), left atrial appendage, and esophagus were constructed using the ICE catheter and 3D mapping (CARTO 3 system, Biosense Webster). A contact force-sensing catheter (SmartTouch Surround Flow or SmartTouch ablation catheter, Biosense Webster) was advanced into the right atrium and CS to construct a 3D EAM shell and create a matrix for visualization. A steerable decapolar catheter (DecaNav, Biosense Webster) was advanced into the CS under EAM guidance. Double/single transseptal puncture was performed under ICE guidance without fluoroscopy. A multielectrode mapping catheter (Lasso or Pentaray catheter, Biosense Webster) and an ablation catheter were advanced into the left atrium, where the geometries of the PVs and atria were verified and refined by EAM with the Lasso or Pentaray catheters. PV isolation (PVI) was performed with the use of an STSF or ST catheter, and a bidirectional block confirmed by the Lasso or Pentaray catheter. A steerable sheath was recommended to improve the stability of the ablation catheter. A power setting of 30 to 45 W was utilized, with irrigation flow of 8 to 30 mL/min, adjusted according to catheter tip temperature feedback. The target contact force was 5 to 15 g. PVI was performed based on the ablation index (AI), with an AI of 500 recommended for the anterior, superior, and inferior walls, and an AI of 300 to 350 recommended for the posterior wall. If ablation was performed near the esophagus or other critical structures, the operator could lower the target AI value at their discretion. After the isolation of each ipsilateral PV, a 20-minute observation period was utilized to detect acute PV reconduction, which was treated with ablation. The procedure was concluded on achievement of electrical isolation of all PVs. Further ablation might be performed, at the operator’s discretion, if non–PV-related atrial arrhythmia occurred, for example, atrial tachycardia (AT) from the superior vena cava, mappable AT, or atrial flutter. If AF was sustained after PVI, cardioversion could be considered. If sinus rhythm could not be restored, even by cardioversion, further ablation could be considered at the operator’s discretion. Fluoroscopy could be used at any step, if necessary, but was not encouraged. The original intention of the protocol was to achieve zero-radiation ablation. Repeated quantitative assessments of pericardial effusion were performed, at any time necessary, during and at the end of the procedure.

### Traditional approach group

The ICE catheter was not used. Baseline cardiac contraction was assessed by x-ray at the beginning of the procedure. Double or single transseptal puncture was performed under fluoroscopic guidance. All geometries were constructed by multielectrode mapping catheters. PVI was achieved using an STSF or ST catheter, guided by the CARTO system. A reassessment was performed whenever necessary during the procedure and afterwards to assess cardiac contraction and to exclude a clinically significant pericardial effusion. Other procedural details were the same, as described earlier in the text, in the minimized-fluoroscopy group.

### Endpoints

#### Primary endpoints

The primary efficacy endpoint was freedom from AF recurrence (without antiarrhythmic medication) after ablation, assessed through a time-to-event analysis of the first clinical recurrence recorded after 90 days following the initial ablation. The primary safety endpoint was a composite of death from any cause, stroke or transient ischemic attack, and other serious adverse events.

#### Secondary endpoints

The secondary endpoints were the duration of lead apron use, accumulative radiation exposure, total procedural duration, and PVI time.

#### Follow-up

Patients were scheduled for follow-up visits at 3, 6, 9, and 12 months postprocedure, with subsequent visits every 6 months. At each visit, the patient's symptoms, electrocardiogram (ECG), and Holter monitoring data were collected to assess any AF recurrence. In addition, patients were equipped with a mobile ECG monitor for weekly transmission of heart rhythm data and immediate reporting in case of symptomatic episodes. The initial 3 months postprocedure were regarded as a blanking period, during which antiarrhythmic medications could be administered but were expected to be discontinued afterwards. A recurrence of AF, atrial flutter, or AT was identified if it lasted longer than 30 seconds and was confirmed by ECG, Holter monitor, or mobile ECG monitoring. Although the original study protocol planned a 12-month follow-up for all patients, the COVID-19 pandemic made it difficult to complete regular follow-up visits as scheduled. Therefore, the follow-up plan was adjusted based on the actual situation, and the analysis was conducted using the median follow-up time of 12 months. Time-to-event methods were applied to properly handle censored data.

### Statistical Analysis

Continuous variables were expressed as mean ± SD, and comparisons between groups were evaluated using Student’s *t*-test. Non-normally distributed variables were expressed as interquartile ranges (Q1-Q3) and were compared by Mann-Whitney *U* test. Categorical variables were presented numerically and as percentages, and comparisons between groups were assessed using the chi-square test or Fisher exact test for a small number of events. A log-rank test was used to compare AF recurrence between the 2 groups. The Kaplan-Meier method was used to construct curves showing AF recurrence at all time points in the 2 groups. *P* values <0.05 were considered to indicate statistical significance. The study aimed to verify that non/minimized-fluoroscopy ablation is not inferior to the conventional approach in terms of the primary endpoint. A Cox analysis was constructed to calculate the HR. Noninferiority was defined as a HR <1.5 based on our trial design.^15^ Statistical analyses were performed using SPSS version 22 software (IBM).

## Results

A total of 448 patients were enrolled, with 225 patients in the traditional approach group and 223 patients in the non/minimized-fluoroscopy group. The study flow and patient dropout details are shown in the Appendix. The baseline characteristics were generally very similar in both groups (Table 1). There was no significant difference in the CHA_2_DS_2_-VASc score between the traditional and non/minimized-fluoroscopy groups (1 [Q1-Q3: 1-2] vs 2 [Q1-Q3: 1-3]; *P* = 0.202). However, patients in the non/minimized-fluoroscopy group had higher CHA_2_DS_2_-VASc scores even though allocation was randomized.

**Table 1:** Characteristics of the Patients at Baseline

	Non/Minimized-Fluoroscopy Group (n = 223)	Traditional Approach Group (n = 225)	*P* Value
Age, y	59.1 ± 10.1	58.7 ± 10.0	0.531
>65 y	68 (27.1)	61 (30.5)	0.493
Male	139 (62.3)	126 (56.0)	0.205
BMI	25.1 ± 3.4	24.8 ± 3.3	0.244
Medical history			
Heart failure	6 (2.7)	7 (3.1)	0.864
Hypertension	112 (50.2)	98 (43.6)	0.187
Diabetes	30 (12.5)	29 (12.9)	0.971
CAD	26 (11.7)	37 (16.4)	0.187
Stroke or TIA	25 (11.2)	16 (7.1)	0.180
CHA_2_DS_2_-VASc score	2 (1-3)	1 (1-2)	0.202
0	46 (20.6)	46 (20.4)	1.0
1	63 (28.3)	70 (31.1)	0.576
2	46 (20.6)	60 (26.7)	0.164
>2	68 (30.5)	49 (21.8)	0.046
LA, mm	36.6 ± 5.5	36.3 ± 4.7	0.529
EF, %	65.9 ± 6.0	66.3 ± 5.3	0.485

Values are mean ± SD, n (%), or median (Q1-Q3).

BMI = body mass index; CAD = coronary artery disease; EF = ejection fraction; LA = left atrium; TIA = transient ischemic attack.

The procedural data and comparisons between groups are shown in Table 2. This protocol enabled near zero-radiation procedures (mean <1 mGy) in 7 of 15 centers (46.7%). A total of 125 of 223 patients (56.1%) underwent radiation-free AF ablation in the non/minimized-fluoroscopy group. In this study, both the traditional and non/minimized-fluoroscopy groups achieved a 100% success rate in PVI with no statistically significant difference in isolation time for left or right veins. The rates of first-pass isolation for the left and right PVs showed no statistically significant difference. Acute PV conduction recovery was similar between the 2 groups. Non-PV ablations were applied in 21 of 223 patients (9.4%) in the non/minimized-fluoroscopy group and in 28 of 225 patients (12.4%) in the traditional approach group. Including the time to exclude left atrial thrombus and assess pericardial effusion, the non/minimized-fluoroscopy group maintained a procedural duration similar to that of the traditional approach group (120 [Q1-Q3: 99-143 minutes] vs 120.0 [Q1-Q3: 100-147 minutes]; *P* = 0.865).

**Table 2:** Procedural Data and Comparison Between Groups

	Non/Minimized-Fluoroscopy Group (n = 223)	Traditional Approach Group (n = 225)	*P* Value
Procedure time, min	120 (99-143)	120 (100-147)	0.865
PV isolation	223/223	225/225	1.0
PV isolation time, min	41 (34-50)	41 (33-52)	0.903
Isolation time, left PV, min	20 (17-26)	21 (16-25)	0.855
Isolation time, right PV, min	20 (15-26)	20 (15-25)	0.904
First-pass isolation, left PV	81.6	82.6	0.884
First-pass isolation, right PV	80.7	84.8	0.306
Acute PV conduction recovery	42/223	46/225	0.757
Non-PV ablations	21/223	28/225	0.381
X-ray free	125 (56.1)	—	
X-ray dose, mGy	0.0 (0.0-16.0)	50.0 (21.8-90.3)	<0.001
X-ray time, min	0 (0-2)	5 (3-9)	<0.001
Lead operator radiation protection equipment time, min	0 (0-20)	89 (30-120)	<0.001

Values are median (Q1-Q3), n/N, %, or n (%).

PV = pulmonary vein.

Notably, the non/minimized-fluoroscopy technique resulted in a substantial reduction in x-ray dose (0 [Q1-Q3: 0.0-16.0 mGy] vs 50.0 [Q1-Q3: 21.8-90.3 mGy]; *P* < 0.001) and a significant decrease in the duration of radiation protection equipment use by the operators (0 [Q1-Q3: 0-20 min] vs 88.5 [Q1-Q3: 30-120 min]; *P* < 0.001).

**Figure 1:**
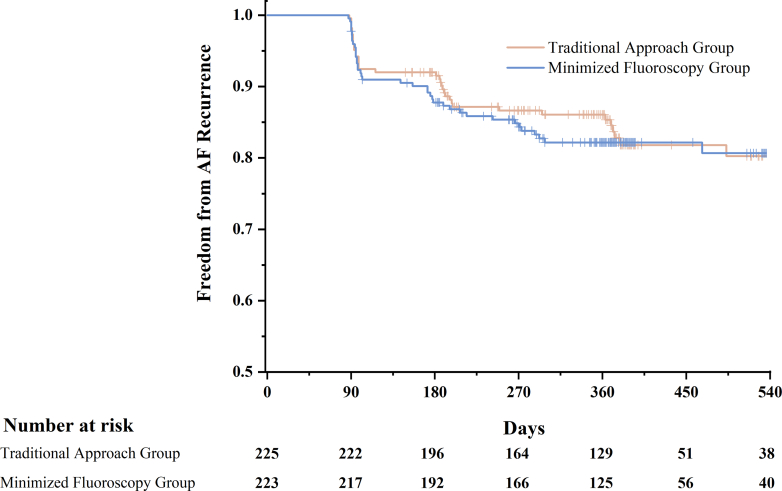
Post-Procedure Event-Free Survival Curve This Kaplan-Meier survival curve compares atrial fibrillation (AF) recurrence-free survival between intracardiac echocardiography–guided non/minimized-fluoroscopy and traditional fluoroscopy-guided catheter ablation. Based on 448 patients with a median follow-up of 12.2 months, the 2 groups showed comparable outcomes (HR: 0.949; 95% CI: 0.774-1.164; *P* = 0.776), highlighting similar efficacy while reducing radiation exposure and protection equipment usage.

A total of 183 of 223 patients (82.5%) in the non/minimized-fluoroscopy group and 189 of 225 patients (84.0%) in the traditional approach group remained free from arrhythmia after a median follow-up period of 12 months, with no difference between the groups (*P* = 0.520). The postprocedure event-free survival curves are shown in Figure 1. The Cox analysis revealed a HR of 0.949 (95% CI: 0.774 to 1.164; *P* = 0.858), demonstrating the noninferiority of the non/minimized-fluoroscopy approach. These results suggest that the non/minimized-fluoroscopy approach significantly minimized radiation exposure without compromising procedural success.

The composite safety endpoint showed no significant difference between the 2 groups, with an RR value of 1.055 (*P* = 0.975). In the non/minimized-fluoroscopy group, there was 1 death secondary to acute myocardial infarction, and 1 case of cardiac tamponade, whereas the traditional approach group experienced 2 cases of stroke (Table 3). The myocardial infarction case occurred more than 7 months after catheter ablation, so we do not consider it to be related to the procedure. The case of cardiac tamponade did not reveal pericardial effusion during the ICE examination before the completion of the procedure. The pericardial effusion appeared relatively delayed, making it difficult to determine the exact step of the procedure during which it occurred. Among the 2 stroke cases, one was a cerebral hemorrhage that occurred 1 year after catheter ablation, whereas the other was a cerebral infarction that occurred on the 18th day after the procedure. In addition, 2 cases of sick sinus syndrome occurred in each group. In the study group, 1 case developed an esophageal ulcer, which healed after active treatment, whereas in the control group, 1 case involved a hospitalization due to syncope. These cases are categorized as “other” in the table (Table 3).

**Table 3:** The Primary Safety Endpoint

	Non/Minimized-Fluoroscopy Group (n = 223)	Traditional Approach Group (n = 225)	*P* Value
AF recurrence–related hospitalization or extended hospital stay	18 (8.1)	17 (7.6)	0.862
Stroke	0	2 (0.9)	0.499
Cardiac tamponade	1 (0.5)	0	0.498
Death	1 (0.5)	0	0.498
Other	3 (1.3)	3 (1.3)	0.999
Sick sinus syndrome	2 (0.9)	2 (0.9)	
Esophageal ulcer	1 (0.5)		
Syncope		1 (0.4)	
Total	23 (10.3)	22 (9.8)	0.975

Values are n (%).

AF = atrial fibrillation.

## Discussion

During EP procedures, fluoroscopy is widely applied for atrial septal puncture and the placement of electrodes and catheters. Adjusted to a 60-minute fluoroscopy exposure, the estimated lifetime risk of additional fatal cancers is reported as 0.07% for females and 0.1% for males.^16^ Wearing a lead apron is a mandatory universal strategy for EP lab staff, including physicians, nurses, and technicians, to prevent radiation exposure. Although lead aprons can effectively protect staff during fluoroscopy, up to 40% of operating room staff may experience orthopedic complications, with symptom severity related to the length of time lead aprons were worn.^5^ In fact, such work-related orthopedic issues may sometimes curtail the professional careers of the affected individuals.

The estimated global prevalence of AF was 50 million in 2020.^17^ Accumulated clinical evidence has led to catheter ablation becoming a first-line treatment option for AF.^1^ On this basis, the annual volume of AF procedures has increased rapidly year on year. The cumulative x-ray doses and lead apron–related orthopedic injuries, particularly in high-volume EP centers, should not be underestimated.

Efforts have been made to reduce the x-ray doses in EP procedures. EAM Systems create detailed 3D maps of cardiac anatomy and electrical activity, and have been instrumental in reducing fluoroscopy time though fluoroscopy cannot be completely avoided. ICE has increasingly been utilized during procedures in recent years. It provides real-time imaging of cardiac structures without radiation and can replace x-rays in most cases.^18^ Studies have shown its effectiveness in guiding transseptal puncture and catheter placement, significantly reducing radiation exposure.^19^^,^^20^ However, ongoing concerns about the safety and efficacy of low radiation techniques, compared with traditional fluoroscopy-guided ablation, remain.

This is the first multicenter, prospective, randomized controlled study of non/minimized-fluoroscopy ablation in patients with paroxysmal AF, as shown in the Central Illustration. The non/minimized-fluoroscopy protocol was successfully implemented in all 15 participating centers, enabling near zero-radiation procedures in 7 centers and a statistically significant reduction in fluoroscopy time and duration of radiation protection equipment wearing in the other centers. Inferior vena cava obstruction cases did not occur in this study. The primary reason for using fluoroscopy is concern about effectively managing potential emergencies in its absence. Due to the study design, data on fluoroscopy time and radiation exposure specifically before and after the transseptal puncture were not collected. However, it has been shown that, in the process of promoting zero-fluoroscopy procedures, fluoroscopy time could be significantly reduced as the number of cases per center increased.^21^ It is believed that through the learning curve and exchange of experience, this protocol can be safely and effectively implemented for zero-fluoroscopy ablation in most centers, even in those lacking experience with zero-fluoroscopy procedures.

**Central Illustration:**
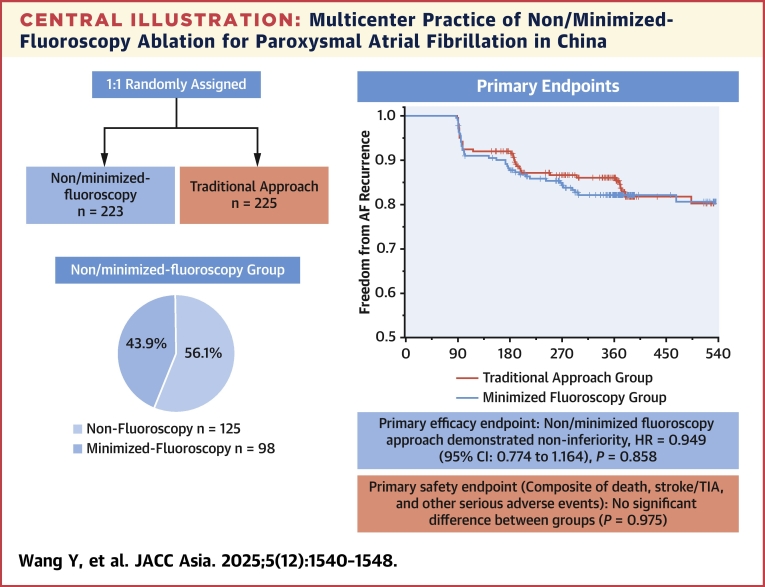
Multicenter Practice of Non/Minimized-Fluoroscopy Ablation for Paroxysmal Atrial Fibrillation in China This illustration provides an overview of the multicenter prospective randomized controlled trial evaluating intracardiac echocardiography–guided non/minimized-fluoroscopy catheter ablation in patients with paroxysmal atrial fibrillation (AF) across 15 centers in China. TIA = transient ischemic attack.

In this study, patients were equipped with a mobile ECG monitor, with scheduled weekly ECG checks or additional checks when symptoms occurred, providing strict monitoring of arrhythmias. Non/minimized-fluoroscopy AF ablation, while maintaining success rates, safety profiles, and very similar procedure times, significantly reduced radiation exposure to both patients and clinical staff, and decreased the duration of lead apron usage, potentially improving the working conditions of health care personnel, reducing occupation-related harm, and prolonging their professional careers. The multicenter practice of the non/minimized-fluoroscopy AF ablation protocol was feasible, and its safety, and efficacy, were demonstrated. Although this was not a real-world study as centers were selected, it merits more widespread adoption.

### Study limitations

In the absence of x-rays, the inability to use an esophageal temperature probe raises concerns. However, esophageal temperature monitoring devices are currently unavailable in China, and their effectiveness in preventing esophageal injury remains inconsistent.^22^ Under ICE, the relationship between the esophagus and the pulmonary veins is usually clearly visible. In this study, esophageal protection was instead achieved by reducing the ablation index at the posterior wall. Given the low risk of esophageal fistula, widely adopted postoperative transesophageal endoscopic evaluation was not performed.

Two centers enrolled a relatively small number of patients (one with 2 cases and the other with 6 cases), which suggests that these centers might still be within the learning curve for the non/minimized-fluoroscopy protocol. Nevertheless, all 15 participating centers are highly experienced electrophysiology institutions with substantial expertise in catheter ablation, with annual AF procedure volumes ranging from 160 to 8,500 cases. All centers also have adequate experience in zero-fluoroscopy atrial fibrillation ablation. The extensive pretrial training and standardization efforts ensured that the protocol could be effectively implemented across all centers. In practice, this protocol demonstrated good operability, even in centers with relatively fewer enrolled cases.

Higher baseline CHA_2_DS_2_-VASc scores were observed in the non/minimized-fluoroscopy group compared to the traditional group (CHA_2_DS_2_-VASc >2; *P* = 0.046), which was entirely attributable to random allocation. Importantly, all other baseline characteristics were well-balanced between the groups. Previous studies have demonstrated that higher CHA_2_DS_2_-VASc scores are associated with an increased risk of atrial fibrillation recurrence and adverse events. Despite this inherent disadvantage in the non/minimized-fluoroscopy group, the noninferiority design of this study provides strong support for the validity of our primary findings. The demonstration of noninferiority in the non/minimized-fluoroscopy approach, even in the context of a higher baseline CHA_2_DS_2_-VASc score, reinforces the robustness of the conclusions. To further minimize such imbalances in future research, stratified randomization based on key baseline characteristics, such as CHA_2_DS_2_-VASc scores, should be considered.

Follow-up duration varied due to the unanticipated impact of the COVID-19 pandemic. A total of 15 of 448 patients (3.3%) had dropped out by 6 months, increasing to 54 patients (12.1%) by 12 months. However, time-to-event analysis and the use of portable ECG monitoring helped ensure the robustness of efficacy assessment.

As the first multicenter randomized controlled trial on non/minimized-fluoroscopy AF catheter ablation, this study did not evaluate preprocedural AF burden due to its design. Moreover, patients with persistent AF, recurrent AF, advanced age, or heart failure with reduced ejection fraction were not included, and pulsed field ablation was not performed. Future studies on zero-fluoroscopy techniques will aim to include a broader patient population.

## Conclusions

ICE-combined non/minimized-fluoroscopy AF ablation was noninferior in effectiveness compared to traditional AF ablation, with no significant difference in safety endpoints. The multicenter practice demonstrated the safety and efficacy of non/minimized-fluoroscopy AF ablation, indicating its potential of widespread adoption.

## Funding Support and Author Disclosures

This study was supported by Biosense Webster. The authors have reported that they have no relationships relevant to the contents of this paper to disclose.
