# Supplementary material for: Multicenter Practice of Non/Minimized Fluoroscopy Ablation for Paroxysmal AF in China: The PAF-ICE Trial
Source: JACC Asia. 2025 Sep 5;5(12):1540–8. doi: 10.1016/j.jacasi.2025.07.011 (PMC12794002; doi:10.1016/j.jacasi.2025.07.011)
Supplement: Supplemental Material [file mmc1.docx]

Appendix I

Enrollment process of PAF-ICE

Appendix II

Estimated Dropout Rates at Follow-up Time Points

| Follow-up | Dropouts | Estimated Dropout Rate |
| --- | --- | --- |
| 3 months | 0 patients | 0.0% |
| 6 months | 15 patients | 3.3% |
| 9 months | 29 patients | 6.5% |
| 12 months | 54 patients | 12.1% |
